# Characterization and prognosis of patients with hepatocellular carcinoma (HCC) in the non-cirrhotic liver

**DOI:** 10.1186/1471-230X-14-117

**Published:** 2014-07-03

**Authors:** Kerstin Schütte, Christian Schulz, Janine Poranzke, Kai Antweiler, Jan Bornschein, Tina Bretschneider, Jörg Arend, Jens Ricke, Peter Malfertheiner

**Affiliations:** 1 Department of Gastroenterology, Hepatology and Infectious Diseases, Otto-von-Guericke University Magdeburg, Leipziger Str. 44, Magdeburg 39120, Germany; 2 Institute for Biometry and Medical Informatics, Otto-von-Guericke University, Magdeburg, Germany; 3 Department of Radiology and Nuclear Medicine, Otto-von-Guericke University, Magdeburg, Germany; 4 Department of General, Visceral and Vascular Surgery, Otto-von-Guericke University, Magdeburg, Germany

**Keywords:** Hepatocellular carcinoma, Prognosis, Liver cirrhosis, Non-cirrhotic liver, NAFLD

## Abstract

**Background:**

HCC predominantly develops in the condition of chronic inflammation that has led to liver cirrhosis. A small proportion of patients with HCC is diagnosed in the non-cirrhotic liver (NCL). Data on patients with HCC in NCL in advanced stages are scarce.

**Methods:**

A retrospective analysis was performed comparing 93 patients with HCC in NCL to 571 patients with HCC in liver cirrhosis (LC) with respect to clinical and demographic characteristics. Also factors influencing survival in patients with HCC in NCL were analyzed.

**Results:**

Patients with HCC in NCL were diagnosed at older age and in more advanced tumor stages than patients with LC. More than 25% of patients with HCC in NCL presented with extrahepatic metastases. Only a minority of patients with HCC in NCL lacked any sign of hepatic damage. Risk factors for LC and risk factors for NAFLD are present in the majority of patients with HCC in NCL. The BCLC classification corresponded with the survival of patients with HCC in NCL although the therapeutic options differ from those for patients with liver cirrhosis.

**Conclusions:**

It will be one of the major challenges in the future to awake awareness of carrying a risk of hepatic malignancies in patients with chronic liver diseases apart from liver cirrhosis, especially in NAFLD. Surveillance programs need to be implemented if these are cost-effective.

## Background

The incidence of hepatocellular carcinoma (HCC) is constantly rising throughout the world with the majority of cases in Asia and Africa due to the high prevalence of hepatitis B virus (HBV) infection [1]. Although Europe is still considered to be a low incidence area, the incidence of HCC in Germany has risen [2] to 6.2 cases/100 000/year with a high mortality of 5.2/100 000/year [3]. HCC develops predominantly in the condition of chronic inflammation evolving into liver cirrhosis (LC) [2]. Therefore liver function in addition to tumor stage and patient related factors has a major impact on treatment decision and prognosis in case of HCC.

The proportion of patients with HCC diagnosed in a non-cirrhotic liver (NCL) varies throughout different geographic regions of the world ranging from 7% to 54% and depends strongly on the leading risk factor for hepatocarcinogenesis [4]. In Western countries, 15%-20% of HCCs are diagnosed in the absence of LC [1,5,6]. Most reports on these patients are from surgically treated cohorts of patients that have undergone curative resection with an obvious selection bias. Data on patients with HCC in NCL in more advanced stages with respect to clinical features and factors influencing survival are scarce in Europe.

Strikingly, previous studies reveal a lower male preponderance of HCC in NCL than in LC. The three main risk factors for HCC (HBV or HCV infection and alcohol abuse) are less frequent than in patients with LC. Patients with HCC in NCL present at more advanced tumor stages than patients with HCC in LC [4,7] because tumors are generally detected when the disease has become symptomatic. The reason for this is that HCC in LC are frequently detected during surveillance ultrasound. However, a larger proportion of patients with HCC in NCL can be treated with curative intent because hepatic resections without the risk of postoperative liver failure are more likely. The absence of advanced underlying chronic liver disease leads to the fact that the tumor burden is the most significant factor influencing survival among further tumor-related, demographic and etiological factors [4,5].

## Methods

Aiming at a clinical characterization of patients with HCC in NCL, a retrospective analysis in a large single-center cohort in Germany was performed. Patients with HCC in NCL were compared to patients with HCC in LC with respect to demographic and clinical characteristics. Additionally, factors influencing the survival of patients with HCC in NCL were analyzed.

The medical records of 714 patients diagnosed and treated with HCC at the University Hospital of Magdeburg between February 1994 and January 2013 were analyzed retrospectively. The analysis included patients who were referred to the Department of Gastroenterology, Hepatology and Infectious Diseases, to the Department of Surgery or to the Department of Radiology and Nuclear Medicine. After excluding patients in whom sufficient data was not available for the purpose of this study, 664 patients were included into the final analysis. Of these, 571 were diagnosed with HCC in LC and 93 patients (14.01%) suffered from HCC in a non-cirrhotic liver.

The retrospective analysis was performed following the guidelines of the Declaration of Helsinki and approved by the ethical review committee of the Otto-von-Guericke University Magdeburg, Germany.

### Epidemiological data

Age, gender, height, weight, calculation of body mass index (BMI) and performance status at the time of diagnosis were recorded.

Liver cirrhosis was either diagnosed histologically or by typical clinical signs, i.e. findings consistent with portal hypertension (enlarged spleen, ascites, esophageal or gastric varices or portal hypertensive gastropathy) combined with sonographical findings. The diagnosis of a non-cirrhotic liver was based on reports of the histopathological evaluation of liver tissue obtained by surgery at time of the resection of HCC or obtained by biopsy from the non-tumor-tissue at the time of diagnosis of HCC. Patients without histological sampling were classified as non-cirrhotic if they were completely free of any evidence of cirrhosis based on clinical, laboratory and radiological findings.

Liver cirrhosis was considered to be a consequence of chronic alcohol abuse if the patient had reported a consumption of more than 60 g alcohol/day or if a history of chronic alcohol abuse was documented in the past medical history. Data on the status of hepatitis B viral infection was available for all and on hepatitis C virus infection for 643 patients.

Information on prevalence of diabetes mellitus as a metabolic risk factor was available for 427 patients. NAFLD was diagnosed by criteria for steatosis in transabdominal ultrasound in the absence of other possible causes of liver disease.

### Serum parameters

To diligently characterize underlying chronic liver disease with respect to liver function, inflammatory activity and etiology, a panel of parameters on clinical chemistry were extracted from the medical records. These included levels of bilirubin, albumin, quick, prothrombin time, alkaline phosphatase, gamma-glutamyl transferase, alanine aminotransferase, aspartate aminotransferase, creatinine, hemoglobin, platelet count, urea nitrogen and protein. If available, levels of ferritin, transferrin saturation, immunoglobulin (Ig) G, IgA, IgM, coeruloplasmin, antinuclear antibodies, antibodies against soluble liver antigen, anti-liver kidney microsomal antigen and alpha-1-antitrypsin were analyzed, too. Additionally, the level of alpha-fetoprotein (AFP) at the time of diagnosis was evaluated. All serum parameter values were classified according to a specified threshold for explorative analyses on parameters’ impact on survival.

### Tumor stage

Radiological reports were used for the assessment of tumor stage. Number and size of HCC nodules as well as the presence of distant metastases including lymph node involvement and information on the patency of the portal vein were recorded. The Barcelona-Clinic Liver Cancer (BCLC) stage was defined respecting tumor stage, liver function as assessed by Child-Pugh-score in patients with LC and clinical performance status of the patient [8]. Additionally, the CLIP score was rated for all patients if possible [9,10].

### Survival data

In 397 cases the exact date of death was taken from the medical records. For the remaining patients, data on survival was censored at the time of the last documented contact to the patient. Thus, the length of survival was calculated from the date of HCC diagnosis to the date of death or to the date of the last documented contact, respectively.

### Statistical analysis

All statistical analyses were performed using IBM SPSS Statistics 21.0.0 (IBM Corporation, New York, N.Y., USA). Results for numerical data are given as median together with minimum and maximum of the sample (i.e. range). For categorical data, results are given as absolute numbers with percentage. For comparison of categorical data, Chi-Square-tests were applied. Mann–Whitney U–tests were used for testing homogeneity of independent samples in continuous data. For the analysis of individual factors influencing survival Cox regression analyses were performed. Factors that were shown to have a significant input on patients’ survival at univariate analysis were included into a multivariate Cox regression analysis except for the two staging systems (CLIP score and BCLC score) that are influenced by several of the clinical and tumor related factors. Median survival times that were also compared by log-rank tests and Kaplan-Meier curves are given for single influencing factors. All tests were carried out two-sided. The level of significance was set to 0.05 without adjusting for multiplicity. The analysis has to be regarded as explorative.

## Results

**Figure 1:**
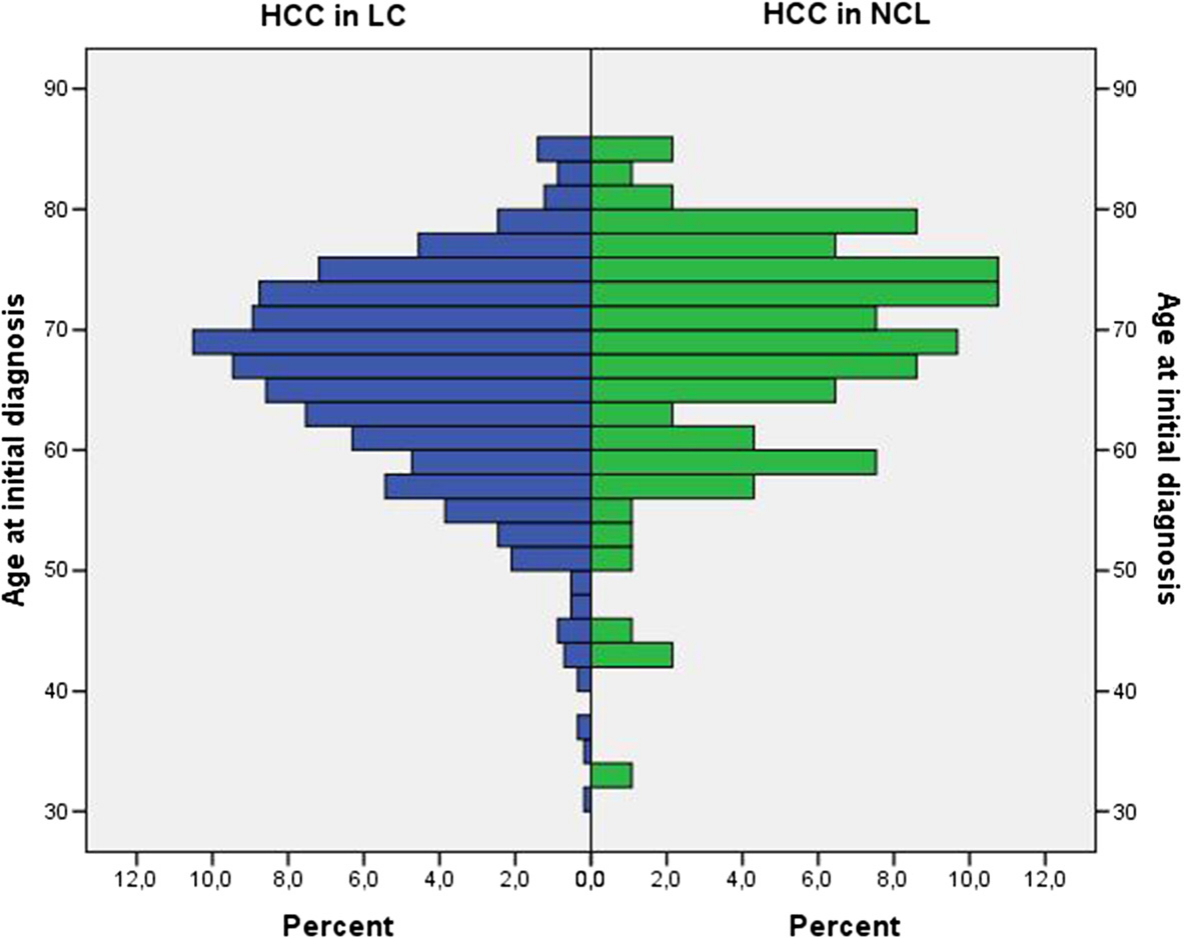
Age distribution of patients with HCC in NCL and LC at initial diagnosis.

The demographic characteristics of patients analyzed are summarized in Table 1. Patients with LC and HCC were diagnosed at a median age of 66 years (range 31–85 years) while patients with HCC in NCL were diagnosed at a statistically significant older age (median age 69 years (range 32–85 years), p = 0.004) (Figure 1).

**Table 1:** Patients’ demographic characteristics

**Parameter**		**HCC in NCL; n = 93**		**HCC in LC; n = 571**		**p**
		**n/median**	**%/range**	**n/median**	**%/range**	
Gender	Male	68	73	468	82	**0.045**
	Female	25	27	103	18	
Age		69	32-85	66	31-85	**0.004**
BMI		24.68	19.18-38.5	27.1	18.07-46.30	**<0.001**
ECOG status		n = 79		n = 428		0.432
	0	37	46.84	195	45.56	
	1	30	37.97	148	34.58	
	2	12	15.19	72	16.82	
	3 or 4	0	0	13	3.04	
Diabetes mellitus	Yes	42 (n = 59)	71.2	308 (n = 368)	83.7	**0.020**
History of nicotine abuse	Yes	22 (n = 55)	40.0	110 (n = 231)	47.6	0.308
Etiology	Alcohol	14	15.05	285	49.91	**0.008**
	Viral	7	7.53	91	15.94	
	NAFLD	6	6.45	37	6.48	
	Other	13	13.98	93	16.29	
	Unknown	53	56.99	65	11.38	

p-values indicating a statistical significance are printed in bold.

Of 571 patients with HCC in LC, 103 were female, resulting in a male to female ratio of 4.5:1 while the proportion of female patients was larger in patients with HCC in NCL with a male to female ratio of 2.7:1 (p = 0.045). Female patients were diagnosed with HCC in NCL at a median age of 66 years while male patients presented at a median age of 71 years (p = 0.383). No statistically significant differences between patients with HCC in NCL and patients with HCC in LC were depicted with respect to their clinical performance status at the time of diagnosis.

When analyzing potential underlying liver diseases or risk factors for the development of HCC, significant differences between the two cohorts were apparent. While in a significant proportion of patients with LC alcohol was identified as causative factor, alcoholic liver disease played a minor role in patients with HCC in NCL and was identified in 15.05% of patients. Viral hepatitis was diagnosed in 7.53% of patients with HCC in NCL (HCV n = 3; HBV n = 4). NAFLD was diagnosed in 6 patients (6.5%). Of these 6 patients, 5 suffered from diabetes mellitus, 5 had been diagnosed with arterial hypertension and 4 presented with a BMI > 25 kg/m^2^. With respect to all patients with HCC in NCL, the BMI exceeded 25 kg/m^2^ in 31 (33.3%) patients, 8 patients (8.6%) were obese and presented with a BMI exceeding 30 kg/m^2^. A significant proportion of patients with HCC in NCL had been diagnosed with diabetes mellitus (n = 42, 45.16%), in 27 patients (29%) a clinical diagnosis of steatosis of the liver had been established before. In the majority of patients (n = 53; 56.99%) with HCC in NCL no underlying chronic liver disease was identified, but in this subgroup of patients the prevalence of metabolic risk factors was also high: 37.74% suffered from diabetes mellitus, 58.5% from arterial hypertension and 22.64% presented with a BMI ≥ 25 kg/m^2^.

A histopathological report regarding the tumor tissue was available in 87 (93.5%) patients. In 21 patients with HCC in NCL the histopathological evaluation of non-neoplastic liver tissue revealed fibrosis of different degrees, in 18 patients fatty liver disease was diagnosed, in 8 patients no fibrosis was detected in non-neoplastic liver tissue.

A comparison of tumor stage at diagnosis revealed that patients with HCC in NCL were diagnosed in more advanced tumor stages than patients with HCC in LC. More than half of the patients with HCC in NCL were in BCLC stage C (51.6% compared to 42.4% of patients with HCC in LC), every third patient was diagnosed in intermediate stage (BCLC B, 34.4% in comparison to 29.6% of patients with HCC in LC). Tumor related characteristics of the two cohorts are summarized in Table 2. Although diagnosed more frequently as uninodular disease (49.46%) than in patients with LC, HCC in NCL were larger than those in LC and were diagnosed more frequently with extrahepatic metastases. The prevalence of portal vein thrombosis was equal between the two cohorts. Ascites was more frequent in patients with HCC in LC.

**Table 2:** Tumor related characteristics of the two cohorts

**Parameter**		**HCC in NCL; n = 93**		**HCC in LC; n = 571**		**p**
		**n/median**	**%/range**	**n/median**	**%/range**	
Number of nodules		n = 93		n = 569		0.053
	1	46	49.46	213	37.43	
	2	5	5.38	60	10.54	
	≥3	42	45.16	296	52.02	
Size of largest nodule (cm)		9.1	2-20	6.5	1-22	**0.002**
Distribution of nodules	Left liver lobe	24	25.81	184	32.22	0.123
	Right liver lobe	34	36.56	157	27.50	
	Both liver lobes	28	30.11	207	36.25	
	No data	7	7.52	23	4.03	
Portal vein thrombosis		n = 86		n = 517		0.732
	Yes	27	31.4	172	33.27	
	No	59	69.6	345	66.73	
Extrahepatic metastasis		n = 86		n = 508		**0.028**
	Yes	32	37.21	131	25.79	
	No	54	62.79	377	74.21	
Ascites		n = 84		n = 535		**< 0.001**
	Yes	17	20.24	237	44.30	
	no	67	79.76	298	55.70	
BCLC stage		n = 93		n = 571		**0.006**
	A	13	14	100	17.5	
	B	32	34.4	169	29.6	
	C	48	51.6	242	42.4	
	D	0	0	60	10.5	
CLIP score		n = 82		n = 483		**0.015**
	0	14	17.07	60	12.42	
	1	20	24.39	111	22.98	
	2	16	19.51	103	21.33	
	3	25	30.49	88	18.22	
	4	5	6.1	59	12.22	
	5	2	2.44	52	10.77	
	6	0	0	10	2.07	

p-values indicating a statistical significance are printed in bold.

The proportion of patients with serum AFP concentration exceeding 200 ng/ml was almost similar between the two groups (33.3% in NCL vs 37.0% in LC).

In the laboratory work-up of the two cohorts (Table 3) statistically significant differences were apparent with respect to liver function at time of diagnosis of HCC. Patients with HCC in LC showed statistically significant elevated concentrations of bilirubin, ASAT, GGT and IgA as well as prolonged PTT in comparison to patients with HCC in NCL. Concentrations of parameters mirroring liver synthesis capacity (quick, albumin) and platelets were significantly lower in patients with HCC in LC compared to non-cirrhotic patients. All other parameters evaluated did not show statistically significant differences.

**Table 3:** Laboratory work-up of patients with HCC in NCL in comparison to patients with HCC in LC

**Parameter (SI-units)**	**Patients with HCC in NCL**			**Patients with HCC in LC**			**p**
	**n**	**Median**	**Range**	**n**	**Median**	**Range**	
Bilirubin	88	11.5	3.3-497.0	547	20.6	3.3-709.1	**< 0.001**
Quick	89	97.0	18-120	547	86.0	18-120	**< 0.001**
Albumin	81	40.10	15.6-71.0	507	36.1	9.5-72.5	**< 0.001**
ALAT	87	0.75	0.04-7.72	545	0.77	0.12-31.20	0.110
ASAT	86	0.76	0.17-12.11	546	1.11	0.26-25.90	**< 0.001**
GGT	80	2.43	0.19-36.28	518	3.26	0.17-66.51	**0.002**
Alcaline phosphatase	81	3.59	0.38-36.46	506	3.41	0.69-138.69	0.737
Creatinine	87	78.00	45-480	535	77.0	2-557	0.622
Urea nitrogen	83	5.8	2.1-44.2	513	5.9	1.6-35.8	0.437
Ferritin	32	452.5	21-2928	233	356.0	3-7585	0.553
Transferrin saturation	29	25.50	8.0-95.3	166	30.3	4.5-119.0	0.310
Coeruloplasmin	16	0.38	0.29-0.53	112	0.36	0.12-2.31	0.951
Hemoglobin	82	8.2	5.7-18.7	505	8.1	4.1-13.7	0.997
Platelets	81	261.0	84-693	506	166.5	33-799	**< 0.001**
PTT	84	28.5	22.1-60.0	527	31.5	19.2-80.2	**< 0.001**
IgA	15	2.56	0.61-22.80	101	5.42	1.27-43.30	**< 0.001**
IgM	13	0.970	0.5-2.6	96	1.19	0.2-15.5	0.274
IgG	14	13.05	8-28	103	16.30	2-46	0.117
CA 19-9	22	35.5	1.5-7520	226	60.00	<0.5-8483.0	0.057
AFP	85	32.10	1-378813.00	531	62.00	0.6-1460000.00	0.168

ALAT = alanine aminotransferase, ASAT = aspartate aminotransferase, GGT = gamma glutamyl transferase, PPT = partial thromboplastin time, IgA = immunoglobulin A, IgG = immunoglobulin G, AFP = alpha fetoprotein, CA 19–9 = carbohydrate antigen 19–9.

p-values indicating a statistical significance are printed in bold.

In a univariate Cox regression analysis for factors influencing survival of patients with HCC in NCL the presence of portal vein thrombosis, the presence of extrahepatic metastases, concentration of AFP, ASAT and platelets as well as the ECOG performance status of the patient were significant factors. A multivariate analysis of these factors confirmed the presence of extrahepatic metastases and the concentration of platelets as factors significantly influencing survival of patients with HCC in NCL (Table 4).

**Table 4:** Cox regression analysis for factors influencing survival in patients with HCC in NCL

**Variables (SI-units if not indicated otherwise)**	**Univariate analysis**			**Multivariate analysis**		
	**HR**	**95% CI**	***p***	**HR**	**95% CI**	***p***
Age (≤65 years vs > 65 years)	1.598	0.957-2.669	0.073			
Sex (m vs f)	0.777	0.457-1.332	0.352			
BMI (≤30 kg/m^2^ vs > 30 kg/m^2^)	0.573	0.216-1.518	0.263			
Etiology (alcohol vs viral vs other)			0.095			
BCLC stage			**0.014**			
CLIP			**0.031**			
Size of largest nodule (>5 cm vs ≤ 5 cm)	1.991	0.993-3.992	0.052			
Portal vein thrombosis (yes vs no)	1.859	1.066-3.241	**0.029**	1.559	0.676-3.596	0.298
Extrahepatic metastasis (yes vs no)	2.104	1.248-3.549	**0.005**	2.844	1.098-7.368	**0.031**
Ascites	1.181	0.575-2.428	0.651			
Number of lesions (one vs two vs multinodular)			0.204			
Steatosis (yes vs no)	0.576	0.248-1.340	0.200			
AFP	1.0	0.999-1.000	**0.031**	1.0	0.999-1.001	0.323
CA 19-9	1.0	0.999-1.001	0.969			
Hemoglobin	0.963	0.790-1.174	0.709			
ALAT	1.060	0.864-1.302	0.575			
ASAT	1.158	1.000-1.340	**0.050**	0.999	0.629-1.586	0.997
Albumin	0.975	0.941-1.010	0.152			
Bilirubin	1.001	0.997-1.006	0.484			
Alcaline phosphatase	0.993	0.955-1.032	0.724			
GGT	1.021	0.974-1.071	0.386			
Quick	0.990	0.977-1.002	0.112			
PTT	1.057	1.007-1.110	**0.026**	1.057	0.891-1.255	0.524
Platelets	1.002	1.000-1.004	**0.049**	1.004	1.001-1.007	**0.006**
Ferritin	1.0	1.000-1.001	0.455			
Creatinine	0.997	0.992-1.002	0.231			
Urea nitrogen	0.966	0.914-1.020	0.212			
Diabetes mellitus (yes vs no)	1.246	0.564-2.753	0.586			
Nicotine (yes vs no)	1.239	0.632-2.428	0.532			
ECOG status (1 or 2 vs 0)	1.965	1.129- 3.417	**0.017**	1.645	0.681-3.968	0.269
Aim of treatment (palliative vs curative)	1.044	0.616-1.770	0.873			

p-values indicating a statistical significance are printed in bold.

**Figure 2:**
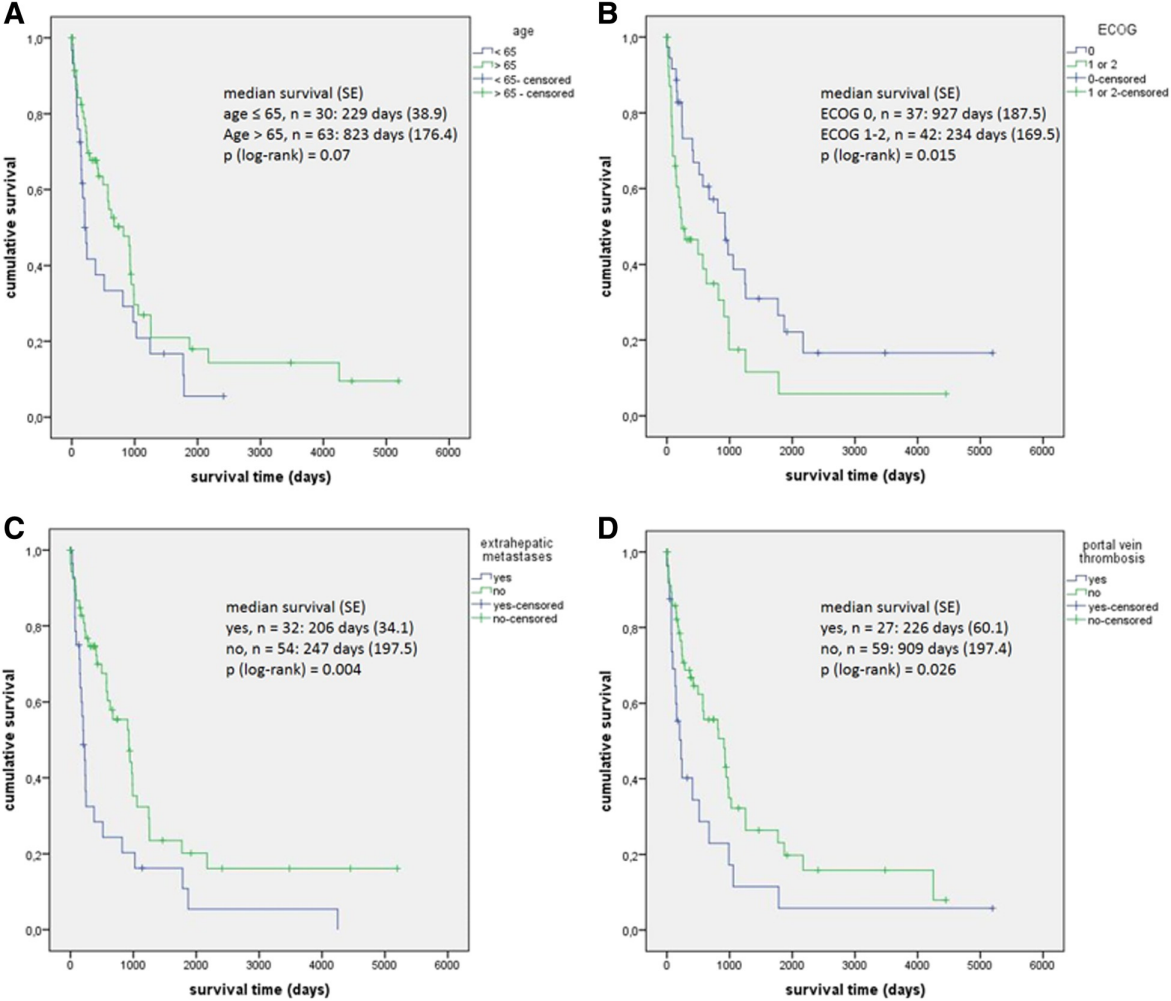
**Univariate explorative analysis of factors influencing survival in patients with HCC in NCL. A** age: ≤ 65 years vs > 65 years. **B** ECOG performance status: 1 or 2 vs 0. **C** presence of extrahepatic metastases yes vs no. **D** presence of portal vein thrombosis yes vs no.

The unadjusted results of further univariate explorative analyses are given in Figures 2 (demographic parameters and tumor related parameters) and 3 (staging systems).

**Figure 3:**
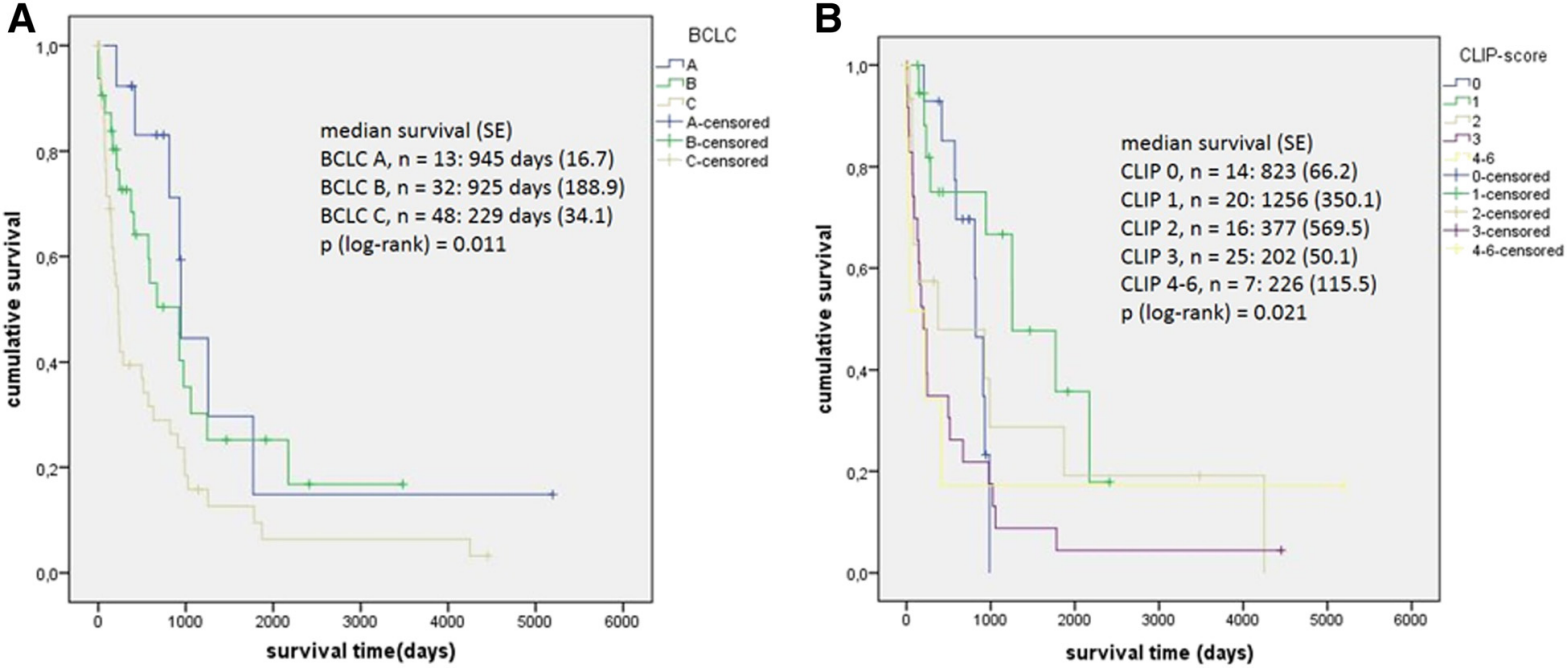
**Univariate explorative analyses of survival of patients with HCC in NCL in dependence of tumor stage according to BCLC and CLIP-score. A** survival (days) according to BCLC score A vs B vs C. **B** survival (days) according to CLIP score 0 vs 1 vs 2 vs 3 vs 4–6.

Regarding the two staging systems applied, the BCLC system correlated better with the survival of patients with HCC in NCL than the CLIP score (Figure 3).

Overall survival according to tumor stage at diagnosis did not differ significantly between patients with HCC in NCL and patients with HCC in liver cirrhosis. Patients diagnosed in BCLC A showed a median survival of 945 days (SD 17) in case of NCL compared to 951 days (SD 176, p = 0.422) in LC. Patients in intermediate stage had a median survival of 925 days (SD 189) in comparison to 501 days (SD 58, p = 0.100) in case of LC. For patients with advanced disease the median survival was 229 days (SD 34) compared to 209 days (SD 15, p = 0.438).

First-line treatment modalities applied in patients with HCC in NCL are summarized in Table 5. In summary, 30.12% of patients underwent treatment in curative intent (resection, liver transplantation or local ablation).

**Table 5:** Treatment modalities applied in patients with HCC in NCL (n = 93) and patients with HCC in LC (n = 571)

		**HCC in NCL**		**HCC in LC**	
	**Treatment modality**	**n**	**%**	**n**	**%**
Curative treatment	Resection	22	23.66	69	12.1
	RFA/thermoablation	5	5.38	27	4.7
	Liver transplantation	1	1.08	8	1.4
**Total**		**28**	**30.11**	**104**	**18.2**
Palliative treatment	Transarterial chemoembolization	14	15.05	128	22.4
	Systemic therapy (chemotherapy or sorafenib)	22	23.66	167	29.3
	Y90-radioembolisation	3	3.23	23	4.0
	HD-interstitial brachytherapy	12	12.90	52	9.1
	Best supportive care	5	5.38	20	3.5
**Total**		**56**	**60.22**	**390**	**68.3**
	No data	9	9.68	77	13.5

## Discussion

Most of the characteristics reported in the literature over the last decades were confirmed in our analysis, but we also identified some differences compared to published data [4,11].

In 14% of our study population HCC has been detected in NCL. Patients with HCC in NCL presented at an older age than patients with HCC in LC and this differs from some of the previous reports [12,13]. We confirmed a larger proportion of female patients with HCC in NCL. In both cohorts of patients, with and without LC, more than 50% of patients initially presented with symptoms and impaired performance status. A previous study from Germany states that 47% of patients present with tumor symptoms leading to the initial diagnosis of HCC, but 86% of patients with HCC in NCL are not restricted in their daily activities at all [5].

For the majority of patients with HCC in NCL the etiology was most likely related to the metabolic syndrome. Even though non-alcoholic fatty liver disease (NAFLD) was diagnosed in a small proportion of patients, factors of the metabolic syndrome were frequent in our cohort, especially in the subgroup of patients in whom no other risk factor was identified. NAFLD, the hepatic manifestation of the metabolic syndrome, is frequently underdiagnosed in the general population but is recognized to be a frequent cause for HCC [14]. The incidence of cirrhosis, on the other hand, in patients with HCC and NAFLD is low in comparison to patients with other causes of HCC [15], and patients with HCC and NAFLD exhibit more features of the metabolic syndrome [15]. Vice versa, about 75% of obese patients suffer from NAFLD [16], and obesity alone has been shown to be a risk factor for HCC with an OR of 1.39 to 4.52 [17-19].

Several cohort studies have demonstrated that diabetes mellitus is an independent risk factor for hepatocellular carcinoma [20]. An analysis within the SEER-database found a 2.9 fold risk for diabetic patients to develop HCC [21]. This risk factor was present in more than 70% of our patients with HCC in NCL.

It is suggested that tumor suppressor genes play an important role in the development of steatosis, induce liver cell damage and therefore promote the formation of HCC in the absence of cirrhosis [15,22,23] in combination with other complex dysregulated mechanisms and pathways in fatty liver disease.

It is striking that, although a direct hepatocarcinogenic role of alcohol has not been proven so far, 15% of patients with HCC in NCL reported a significant intake of alcohol as risk factor for liver disease. This goes along with data previously reported in which 12% to 21.4% of patients with HCC in NCL abused alcohol [4,5,7]. This suggests that cirrhosis is not a condition sine qua non in alcoholic liver disease for HCC development. It is not known whether in these patients the inflammation alone is sufficient or whether fibrosis needs to develop before HCC can arise. A Swedish study demonstrated a 2.4 fold risk for hepatocellular carcinoma in patients with alcohol abuse in comparison to the general population that increased to a 22.4 fold risk in the presence of cirrhosis [24]. An interaction between alcoholic liver disease and other risk factors is likely to occur. Hence, obesity and the metabolic syndrome are factors which favor the progression of alcoholic liver disease and increase hepatocellular carcinoma (HCC) incidence and mortality [25]. Similarly, a synergism has been shown for viral hepatitis and heavy alcohol consumption [26]. Viral hepatitis played a minor role in our cohort.

Since we did not take biopsies from non-tumorous liver tissue as part of the standard work-up of patients with HCC, the number of patients with histology from non-tumorous tissue is rather small. Patients without any hepatic damage of the tissue surrounding the tumor were few and this may indicate the possibility of direct hepatocarcinogenesis in the healthy liver (e.g. adenoma-carcinoma sequence). Most patients showed histological features of chronic liver damage. Chronic liver disease thus may not have been recognized in advance of hepatocellular carcinoma and therefore no surveillance has been offered. This is probably the reason why diagnosis is made in advanced tumor stages in these patients.

Consistent with previous studies almost every second patient with HCC in NCL was diagnosed with a single tumorous nodule but of larger size [11,27]. The proportion of patients with extrahepatic metastases in our cohort exceeded 25% and was strikingly larger than reported in other cohorts [5,28] with up to 15%.

The BCLC staging system, although developed for patients with LC, correlates with patients’ survival. The CLIP score was less suitable to predict survival of patients with HCC in NCL. Patients in intermediate stage HCC according to BCLC in NCL show a trend towards prolonged survival in comparison to patients with LC. This is most likely because curative treatment can be offered to these patients even if the tumor load exceeds the Milan criteria.

Survival of patients with HCC in NCL mainly depends on tumor related factors such as tumor size, existence of satellite lesions, existence of a tumor capsule, vascular invasion, grading, R0 resection and the amount of intraoperative blood transfusions [28-35]. Analyses from mixed surgically and medically treated cohorts of patients identified tobacco consumption, clinical performance status, siderosis of non-tumorous tissue, tumor stage according to BCLC and the initial treatment to be relevant for survival [5,7]. Our study confirms tumor related factors to exert a significant influence on patients’ outcome in univariate explorative analyses (portal vein thrombosis, existence of extrahepatic tumor manifestations). Tumor size and number of intrahepatic HCC nodules had not statistically significant impact on survival. Ongoing hepatic inflammation, mirrored in elevation of ASAT, reduced hepatic synthesis capacity mirrored in prolonged PTT and low platelets (probably a consequence of portal hypertension caused by portal vein thrombosis or by large hepatic tumor load) were negative predictive for survival for patients with HCC in NCL. At multivariate analysis solely the presence of metastases and low platelets were significant factors influencing survival.

Although the proportion of patients who presented at an early tumor stage was rather small, it was evident that in addition to patients in BCLC A some patients at an intermediate tumor stage (BCLC B) also qualified for treatment in curative intent. A modification of current treatment algorithms for patients with HCC in NCL is desirable.

Our study has some limitations due to the retrospective design and the small number of patients in some of the univariate analyses might lead to insignificant findings in the statistical analyses. The mixed cohort of patients does not permit to address surgical aspects.

## Conclusion

HCC in NCL has distinct features compared to HCC in LC. The majority of patients with HCC in NCL present with signs of hepatic damage even in the absence of LC. Risk factors for LC and risk factors for NAFLD are present in the majority of these patients and surveillance programs need to be implemented if these are cost-effective.

In this cohort the proportion of patients with advanced tumor stage and especially with extrahepatic metastases was larger than expected from previously published surgically treated cohorts. The BCLC staging algorithm correlates with patients’ survival. However, therapeutic decisions should not be based on this staging system as in non-cirrhotic patients curative treatment can be offered also to patients with large intrahepatic tumors outside the Milan criteria.

## Competing interests

This work was not supported by any grant or funding source. The authors disclose no potential competing interests.

## Authors’ contributions

KS, CS, JP, JB, TB, JA performed the study. KS and KA performed the statistical analysis. KS, CS and JB wrote the paper. PM and JR supervised and drafted the study and the manuscript and performed a final revision. All authors read and approved the final manuscript.

## Pre-publication history

The pre-publication history for this paper can be accessed here:

http://www.biomedcentral.com/1471-230X/14/117/prepub
